# 2-(1,3-Benzothia­zol-2-ylsulfan­yl)-1-phenyl­ethanone

**DOI:** 10.1107/S1600536809033121

**Published:** 2009-09-12

**Authors:** Hossein Loghmani-Khouzani, Dariush Hajiheidari, Ward T. Robinson, Noorsaadah Abdul Rahman, Reza Kia

**Affiliations:** a Chemistry Department, University of Isfahan, Isfahan 81746-73441, Iran; b Department of Chemistry, University of Malaya, 50603 Kuala Lumpur, Malaysia; c Department of Chemistry, Science and Research Campus, Islamic Azad University, Poonak, Tehran, Iran

## Abstract

In the mol­ecule of the title compound, C_15_H_11_NOS_2_, the 1,3-benzothia­zole ring is oriented at a dihedral angle of 6.61 (6)° with respect to the phenyl ring. In the crystal structure, inter­molecular C—H⋯O inter­actions link the mol­ecules in a herring-bone arrangement along the *b* axis and π–π contacts between the thia­zole and phenyl rings [centroid–centroid distance = 3.851 (1) Å] may further stabilize the structure.

## Related literature

For applications of the title compound in organic synthesis, see: Marco *et al.* (1995 ▶); Fuju *et al.* (1988 ▶); Ni *et al.* (2006 ▶); Grossert *et al.* (1984 ▶); Oishi *et al.* (1988 ▶); Antane *et al.* (2004 ▶). For its biological activity, see: Padmavathi *et al.* (2008 ▶).
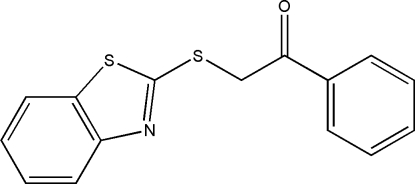


## Experimental

### 

#### Crystal data

C_15_H_11_NOS_2_
*M* *_r_* = 285.37
Orthorhombic, 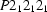

*a* = 5.1060 (1) Å
*b* = 14.6220 (3) Å
*c* = 17.3920 (4) Å
*V* = 1298.49 (5) Å^3^
*Z* = 4
Mo *K*α radiation
μ = 0.40 mm^−1^
*T* = 295 K
0.45 × 0.32 × 0.17 mm

#### Data collection

Bruker SMART APEXII CCD area-detector diffractometer
Absorption correction: multi-scan (*SADABS*; Bruker, 2005 ▶) *T*_min_ = 0.841, *T*_max_ = 0.935
14309 measured reflections
3734 independent reflections
3576 reflections with *I* > 2σ(*I*)
*R*_int_ = 0.024

#### Refinement

*R*[*F* ^2^ > 2σ(*F* ^2^)] = 0.027
*wR*(*F* ^2^) = 0.068
*S* = 1.07
3734 reflections
172 parameters
H-atom parameters constrained
Δρ_max_ = 0.31 e Å^−3^
Δρ_min_ = −0.16 e Å^−3^
Absolute structure: Flack (1983 ▶), 1550 Friedel pairs
Flack parameter: 0.01 (5)

### 

Data collection: *APEX2* (Bruker, 2005 ▶); cell refinement: *SAINT* (Bruker, 2005 ▶); data reduction: *SAINT*; program(s) used to solve structure: *SIR2004* (Burla *et al.*, 2004 ▶); program(s) used to refine structure: *SHELXTL* (Sheldrick, 2008 ▶); molecular graphics: *SHELXTL*; software used to prepare material for publication: *SHELXTL* and *PLATON* (Spek, 2009 ▶).

## Supplementary Material

Crystal structure: contains datablocks global, I. DOI: 10.1107/S1600536809033121/hk2756sup1.cif

Structure factors: contains datablocks I. DOI: 10.1107/S1600536809033121/hk2756Isup2.hkl

Additional supplementary materials:  crystallographic information; 3D view; checkCIF report

## Figures and Tables

**Table 1:** Hydrogen-bond geometry (Å, °)

*D*—H⋯*A*	*D*—H	H⋯*A*	*D*⋯*A*	*D*—H⋯*A*
C2—H2*A*⋯O1^i^	0.93	2.51	3.2299 (17)	135

Symmetry code: (i) 
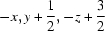
.

**Fig. 1.**
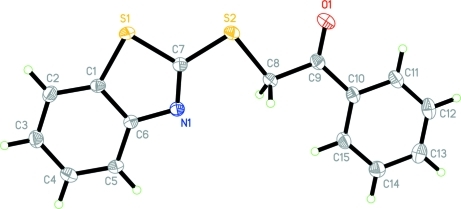
The molecular structure of the title molecule with the atom-numbering scheme. Displacement ellipsoids are drawn at the 40% probability level

**Fig. 2.**
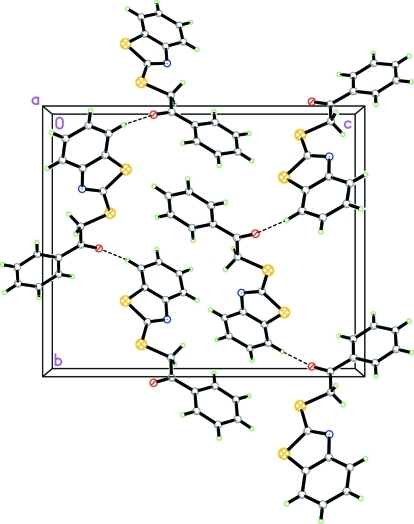
A partial packing diagram. Hydrogen bonds are shown as dashed lines.

## supplementary crystallographic information

### Comment

2-(Benzo[d]thiaazol-2-ylthio)-1-phenylethanone is of great importance in organic synthesis and β-keto-sulfones are a very important group of intermediates as they are precursors for Michael and Knoevenagel reactions and are used in the preparation of acetylenes, allenes, chalcones, vinyl sulfones, polyfunctionalized 4H-pyrans and ketones (Marco *et al.*, 1995; Fuju *et al.*,  1988; Ni *et al.*,  2006). In addition, β-keto-sulfones can be converted into optically active β-hydroxy-sulfones, halomethyl and dihalomethyl sulfones. Halomethyl and dihalomethyl sulfones are very good α-carbanion stabilizing substituents and precursors for the preparation of alkenes, aziridines, epoxides and β-hydroxy-sulfones. Haloalkyl sulfones are useful in preventing aquatic organisms from attaching to fishing nets and ship hulls (Grossert *et al.*,  1984; Oishi *et al.*, 1988; Antane *et al.*,  2004). They also possess other biological properties such as herbicidal, bactericidal antifungal and insecticidal. Recently sulfone-linked heterocycles were prepared and have been showed antimicrobial activity (Padmavathi *et al.*,  2008). We prepared the title compound as a precursor for the synthesis of gem-difluoromethylene-containing heterocycle, and reported herein its crystal structure.

In the molecule of the title compound, (Fig. 1), the benzothiazole ring is oriented with respect to the phenyl ring at a dihedral angle of 6.61 (6)°. In the crystal structure, intermolecular C-H···O interactions (Table 1) link the molecules in herringbone mode along the b axis (Fig. 2), in which they may be effective in the stabilization of the structure. The π–π contact between the thiazole and phenyl rings, Cg1—Cg2^i^, [symmetry code: (i) x - 1, y, z, where Cg1 and Cg2 are centroids of the rings (S1/N1/C1/C6/C7) and (C1-C6), respectively] may further stabilize the structure, with centroid-centroid distance of 3.851 (1) Å.

### Experimental

For the preparation of the title compound, sodium carbonate (4.5 mmol) was added to a stirred solution of 2-mercaptobenzothiazole (3 mmol) in ethanol (15 ml) and water (15 ml) and stirred at room temperature for 30 min. α-Bromoacetophenone (3 mmol) was added to the reaction mixture and stirring was continued for 1h. The reaction was monitored by TLC and after 60 min showed the complete disappearance of the starting material. The reaction mixture was poured into HCl (1M, 100 ml) containing crushed ice (50 g). The product was filtered under vacuum and filtrate washed with ice-cold ethanol (10 ml) and water (10 ml). Recrystalization from petrol ether and filtration gave the title compound (m.p. 387-389 K). ^1^H NMR (400 MHz; CDCl_3_): 8.15-7.75 (m, 4H), 7.55-7.32 (m, 5H), 5.10 (s, 2H). ^13^C NMR (126 MHz; CDCl_3_): 194.2 (C═O), 162.1, 151.8, 135.3, 134.2, 131.1, 126.7, 126.5, 124.7, 124.1, 119.8, 119.6, 37.5. Anal. Calcd. for CHNS: C, 63.13; H, 3.89; N, 4.91. Found: C, 63.07; H, 3.86; N, 4.93.

### Refinement

H atoms were positioned geometrically with C-H = 0.93 and 0.97 Å for aromatic and methylene H atoms, respectively, and constrained to ride on their parent atoms, with U_iso_(H) = 1.2U_eq_(C).

### Crystal data

**Table:**

C_15_H_11_NOS_2_	*D*_x_ = 1.460 Mg m^−^^3^
*M**_r_* = 285.37	Melting point: 388 K
Orthorhombic, *P*2_1_2_1_2_1_	Mo *K*α radiation, λ = 0.71073 Å
Hall symbol: P 2ac 2ab	Cell parameters from 8370 reflections
*a* = 5.1060 (1) Å	θ = 2.3–30.5°
*b* = 14.6220 (3) Å	µ = 0.40 mm^−^^1^
*c* = 17.3920 (4) Å	*T* = 295 K
*V* = 1298.49 (5) Å^3^	Block, pale-yellow
*Z* = 4	0.45 × 0.32 × 0.17 mm
*F*(000) = 592	

### Data collection

**Table:**

Bruker SMART APEXII CCD area-detector diffractometer	3734 independent reflections
Radiation source: fine-focus sealed tube	3576 reflections with *I* > 2σ(*I*)
graphite	*R*_int_ = 0.024
φ and ω scans	θ_max_ = 30.0°, θ_min_ = 2.7°
Absorption correction: multi-scan (*SADABS*; Bruker, 2005)	*h* = −7→6
*T*_min_ = 0.841, *T*_max_ = 0.935	*k* = −20→20
14309 measured reflections	*l* = −24→24

### Refinement

**Table:**

Refinement on *F*^2^	Secondary atom site location: difference Fourier map
Least-squares matrix: full	Hydrogen site location: inferred from neighbouring sites
*R*[*F*^2^ > 2σ(*F*^2^)] = 0.027	H-atom parameters constrained
*wR*(*F*^2^) = 0.068	*w* = 1/[σ^2^(*F*_o_^2^) + (0.0411*P*)^2^ + 0.1348*P*] where *P* = (*F*_o_^2^ + 2*F*_c_^2^)/3
*S* = 1.07	(Δ/σ)_max_ = 0.001
3734 reflections	Δρ_max_ = 0.31 e Å^−^^3^
172 parameters	Δρ_min_ = −0.16 e Å^−^^3^
0 restraints	Absolute structure: Flack (1983), 1550 Friedel pairs
Primary atom site location: structure-invariant direct methods	Flack parameter: 0.01 (5)

### Special details

**Table:**

Geometry. All esds (except the esd in the dihedral angle between two l.s. planes) are estimated using the full covariance matrix. The cell esds are taken into account individually in the estimation of esds in distances, angles and torsion angles; correlations between esds in cell parameters are only used when they are defined by crystal symmetry. An approximate (isotropic) treatment of cell esds is used for estimating esds involving l.s. planes.
Refinement. Refinement of F^2^ against ALL reflections. The weighted R-factor wR and goodness of fit S are based on F^2^, conventional R-factors R are based on F, with F set to zero for negative F^2^. The threshold expression of F^2^ > 2sigma(F^2^) is used only for calculating R-factors(gt) etc. and is not relevant to the choice of reflections for refinement. R-factors based on F^2^ are statistically about twice as large as those based on F, and R- factors based on ALL data will be even larger.

### Fractional atomic coordinates and isotropic or equivalent isotropic displacement parameters (Å^2^)

**Table:**

	*x*	*y*	*z*	*U*_iso_*/*U*_eq_	
S1	0.32503 (6)	0.26829 (2)	0.744608 (17)	0.02052 (8)	
S2	0.01546 (7)	0.10723 (2)	0.799872 (17)	0.02062 (8)	
O1	−0.3471 (2)	−0.02751 (8)	0.84299 (6)	0.0341 (3)	
N1	0.3955 (2)	0.19530 (7)	0.87979 (6)	0.0178 (2)	
C1	0.5575 (2)	0.31781 (8)	0.80443 (7)	0.0173 (2)	
C2	0.7199 (3)	0.39273 (9)	0.79124 (8)	0.0226 (3)	
H2A	0.7112	0.4252	0.7453	0.027*	
C3	0.8936 (3)	0.41738 (9)	0.84818 (8)	0.0240 (3)	
H3A	1.0056	0.4666	0.8402	0.029*	
C4	0.9045 (3)	0.36954 (9)	0.91801 (8)	0.0223 (3)	
H4A	1.0230	0.3876	0.9557	0.027*	
C5	0.7413 (3)	0.29592 (9)	0.93148 (7)	0.0188 (2)	
H5A	0.7477	0.2648	0.9781	0.023*	
C6	0.5668 (2)	0.26908 (9)	0.87415 (6)	0.0159 (2)	
C7	0.2610 (3)	0.18779 (9)	0.81703 (7)	0.0171 (2)	
C8	0.0085 (3)	0.05695 (9)	0.89446 (7)	0.0219 (2)	
H8A	0.1752	0.0279	0.9053	0.026*	
H8B	−0.0207	0.1041	0.9327	0.026*	
C9	−0.2085 (3)	−0.01289 (9)	0.89821 (8)	0.0208 (3)	
C10	−0.2437 (3)	−0.06360 (9)	0.97210 (8)	0.0197 (2)	
C11	−0.4423 (3)	−0.12874 (9)	0.97723 (9)	0.0255 (3)	
H11A	−0.5527	−0.1388	0.9355	0.031*	
C12	−0.4758 (3)	−0.17854 (10)	1.04421 (9)	0.0296 (3)	
H12A	−0.6096	−0.2215	1.0476	0.036*	
C13	−0.3103 (3)	−0.16447 (9)	1.10627 (9)	0.0271 (3)	
H13A	−0.3309	−0.1991	1.1507	0.032*	
C14	−0.1145 (3)	−0.09906 (10)	1.10233 (8)	0.0271 (3)	
H14A	−0.0055	−0.0891	1.1443	0.032*	
C15	−0.0816 (3)	−0.04858 (10)	1.03552 (8)	0.0240 (3)	
H15A	0.0493	−0.0044	1.0329	0.029*	

### Atomic displacement parameters (Å^2^)

**Table:**

	*U*^11^	*U*^22^	*U*^33^	*U*^12^	*U*^13^	*U*^23^
S1	0.02236 (15)	0.02516 (15)	0.01403 (13)	−0.00182 (12)	−0.00251 (11)	0.00181 (11)
S2	0.02335 (15)	0.02168 (14)	0.01685 (13)	−0.00322 (13)	−0.00214 (12)	−0.00344 (11)
O1	0.0360 (6)	0.0339 (5)	0.0323 (5)	−0.0120 (5)	−0.0132 (5)	0.0001 (4)
N1	0.0199 (5)	0.0188 (5)	0.0148 (5)	−0.0008 (4)	0.0011 (4)	−0.0018 (4)
C1	0.0166 (5)	0.0199 (5)	0.0154 (5)	0.0016 (4)	−0.0003 (4)	−0.0011 (4)
C2	0.0233 (6)	0.0220 (6)	0.0224 (6)	0.0001 (5)	0.0012 (5)	0.0051 (5)
C3	0.0223 (6)	0.0193 (6)	0.0303 (7)	−0.0026 (5)	−0.0002 (5)	0.0022 (5)
C4	0.0202 (6)	0.0231 (6)	0.0237 (6)	0.0003 (5)	−0.0041 (5)	−0.0042 (5)
C5	0.0207 (6)	0.0197 (6)	0.0160 (5)	0.0021 (5)	−0.0016 (4)	−0.0006 (4)
C6	0.0168 (5)	0.0164 (5)	0.0144 (5)	0.0018 (5)	0.0012 (4)	−0.0015 (4)
C7	0.0188 (6)	0.0175 (5)	0.0150 (5)	0.0002 (4)	0.0019 (4)	−0.0021 (4)
C8	0.0226 (6)	0.0233 (6)	0.0199 (5)	−0.0065 (6)	−0.0036 (5)	0.0005 (5)
C9	0.0197 (6)	0.0173 (6)	0.0253 (6)	−0.0004 (5)	−0.0011 (5)	−0.0033 (5)
C10	0.0172 (6)	0.0155 (5)	0.0265 (6)	0.0010 (4)	0.0018 (5)	−0.0026 (5)
C11	0.0211 (7)	0.0221 (6)	0.0333 (7)	−0.0042 (5)	−0.0012 (5)	−0.0039 (5)
C12	0.0255 (7)	0.0225 (6)	0.0409 (8)	−0.0054 (6)	0.0072 (6)	−0.0009 (6)
C13	0.0282 (7)	0.0229 (6)	0.0301 (7)	0.0020 (6)	0.0098 (6)	0.0020 (5)
C14	0.0280 (7)	0.0280 (7)	0.0253 (6)	−0.0025 (6)	−0.0006 (5)	0.0005 (6)
C15	0.0206 (6)	0.0228 (6)	0.0285 (6)	−0.0051 (5)	−0.0006 (5)	0.0006 (5)

### Geometric parameters (Å, °)

**Table:**

S1—C1	1.7365 (13)	C5—H5A	0.9300
S1—C7	1.7548 (13)	C8—C9	1.5080 (18)
S2—C7	1.7459 (13)	C8—H8A	0.9700
S2—C8	1.8022 (13)	C8—H8B	0.9700
O1—C9	1.2122 (16)	C9—C10	1.4945 (19)
N1—C7	1.2944 (16)	C10—C11	1.3943 (18)
N1—C6	1.3923 (17)	C10—C15	1.3964 (19)
C1—C2	1.3929 (18)	C11—C12	1.384 (2)
C1—C6	1.4073 (17)	C11—H11A	0.9300
C2—C3	1.3774 (19)	C12—C13	1.386 (2)
C2—H2A	0.9300	C12—H12A	0.9300
C3—C4	1.4026 (19)	C13—C14	1.385 (2)
C3—H3A	0.9300	C13—H13A	0.9300
C4—C5	1.3813 (19)	C14—C15	1.387 (2)
C4—H4A	0.9300	C14—H14A	0.9300
C5—C6	1.3934 (17)	C15—H15A	0.9300
			
C1—S1—C7	88.68 (6)	S2—C8—H8A	109.8
C7—S2—C8	97.66 (6)	C9—C8—H8B	109.8
C7—N1—C6	109.87 (11)	S2—C8—H8B	109.8
C2—C1—C6	121.32 (12)	H8A—C8—H8B	108.3
C2—C1—S1	129.51 (10)	O1—C9—C10	121.56 (13)
C6—C1—S1	109.17 (9)	O1—C9—C8	120.95 (12)
C3—C2—C1	118.08 (12)	C10—C9—C8	117.49 (11)
C3—C2—H2A	121.0	C11—C10—C15	119.22 (13)
C1—C2—H2A	121.0	C11—C10—C9	118.78 (12)
C2—C3—C4	121.17 (13)	C15—C10—C9	121.99 (12)
C2—C3—H3A	119.4	C12—C11—C10	120.20 (14)
C4—C3—H3A	119.4	C12—C11—H11A	119.9
C5—C4—C3	120.77 (12)	C10—C11—H11A	119.9
C5—C4—H4A	119.6	C11—C12—C13	120.13 (13)
C3—C4—H4A	119.6	C11—C12—H12A	119.9
C4—C5—C6	118.93 (12)	C13—C12—H12A	119.9
C4—C5—H5A	120.5	C14—C13—C12	120.25 (13)
C6—C5—H5A	120.5	C14—C13—H13A	119.9
N1—C6—C5	124.71 (11)	C12—C13—H13A	119.9
N1—C6—C1	115.57 (10)	C13—C14—C15	119.76 (14)
C5—C6—C1	119.71 (12)	C13—C14—H14A	120.1
N1—C7—S2	125.63 (10)	C15—C14—H14A	120.1
N1—C7—S1	116.71 (10)	C14—C15—C10	120.42 (13)
S2—C7—S1	117.63 (7)	C14—C15—H15A	119.8
C9—C8—S2	109.28 (9)	C10—C15—H15A	119.8
C9—C8—H8A	109.8		
			
C7—S1—C1—C2	179.53 (13)	C8—S2—C7—S1	−172.57 (8)
C7—S1—C1—C6	0.04 (9)	C1—S1—C7—N1	0.00 (11)
C6—C1—C2—C3	0.86 (19)	C1—S1—C7—S2	177.93 (8)
S1—C1—C2—C3	−178.59 (11)	C7—S2—C8—C9	175.74 (9)
C1—C2—C3—C4	−1.0 (2)	S2—C8—C9—O1	−0.42 (16)
C2—C3—C4—C5	0.2 (2)	S2—C8—C9—C10	179.05 (10)
C3—C4—C5—C6	0.7 (2)	O1—C9—C10—C11	−0.4 (2)
C7—N1—C6—C5	−179.59 (12)	C8—C9—C10—C11	−179.87 (12)
C7—N1—C6—C1	0.07 (15)	O1—C9—C10—C15	178.67 (13)
C4—C5—C6—N1	178.80 (12)	C8—C9—C10—C15	−0.79 (18)
C4—C5—C6—C1	−0.84 (19)	C15—C10—C11—C12	−0.7 (2)
C2—C1—C6—N1	−179.62 (11)	C9—C10—C11—C12	178.35 (13)
S1—C1—C6—N1	−0.07 (13)	C10—C11—C12—C13	−0.6 (2)
C2—C1—C6—C5	0.06 (19)	C11—C12—C13—C14	1.5 (2)
S1—C1—C6—C5	179.61 (10)	C12—C13—C14—C15	−1.0 (2)
C6—N1—C7—S2	−177.78 (9)	C13—C14—C15—C10	−0.3 (2)
C6—N1—C7—S1	−0.04 (14)	C11—C10—C15—C14	1.2 (2)
C8—S2—C7—N1	5.15 (13)	C9—C10—C15—C14	−177.84 (13)

### Hydrogen-bond geometry (Å, °)

**Table:**

*D*—H···*A*	*D*—H	H···*A*	*D*···*A*	*D*—H···*A*
C2—H2A···O1^i^	0.93	2.51	3.2299 (17)	135

Symmetry codes: (i) −*x*, *y*+1/2, −*z*+3/2.
